# Comprehensive Review on the Interactions of Clay Minerals With Animal Physiology and Production

**DOI:** 10.3389/fvets.2022.889612

**Published:** 2022-05-10

**Authors:** Anna Damato, Fabio Vianello, Enrico Novelli, Stefania Balzan, Matteo Gianesella, Elisa Giaretta, Gianfranco Gabai

**Affiliations:** ^1^Department of Comparative Biomedicine and Food Science, University of Padua, Padua, Italy; ^2^Department of Animal Medicine, Production and Health, University of Padua, Padua, Italy

**Keywords:** animal production, cell interactions, clay minerals, gastro-intestinal effects, microbiome

## Abstract

Clay minerals are naturally occurring rock and soil materials primarily composed of fine-grained aluminosilicate minerals, characterized by high hygroscopicity. In animal production, clays are often mixed with feed and, due to their high binding capacity towards organic molecules, used to limit animal absorption of feed contaminants, such as mycotoxins and other toxicants. Binding capacity of clays is not specific and these minerals can form complexes with different compounds, such as nutrients and pharmaceuticals, thus possibly affecting the intestinal absorption of important substances. Indeed, clays cannot be considered a completely inert feed additive, as they can interfere with gastro-intestinal (GI) metabolism, with possible consequences on animal physiology. Moreover, clays may contain impurities, constituted of inorganic micronutrients and/or toxic trace elements, and their ingestion can affect animal health. Furthermore, clays may also have effects on the GI mucosa, possibly modifying nutrient digestibility and animal microbiome. Finally, clays may directly interact with GI cells and, depending on their mineral grain size, shape, superficial charge and hydrophilicity, can elicit an inflammatory response. As in the near future due to climate change the presence of mycotoxins in feedstuffs will probably become a major problem, the use of clays in feedstuff, given their physico-chemical properties, low cost, apparent low toxicity and eco-compatibility, is expected to increase. The present review focuses on the characteristics and properties of clays as feed additives, evidencing pros and cons. Aims of future studies are suggested, evidencing that, in particular, possible interferences of these minerals with animal microbiome, nutrient absorption and drug delivery should be assessed. Finally, the fate of clay particles during their transit within the GI system and their long-term administration/accumulation should be clarified.

## Introduction

Clays are naturally occurring rock or soil materials primarily composed of fine-grained minerals, characterized by high plasticity when hydrated and hardness in the dry form. The most widespread criterion for clay classification is based on their particle size, even though a general agreement has not been achieved (1), and sedimentologists, geologists and colloidal chemists classify as clay materials with particle size smaller than 4, 2, and 1 μm, respectively (2). Clays are readily available in nature, but their properties vary considerably depending on the geological origin and on post-extraction treatments (3, 4). Depending on their structure and physico-chemical properties (particle size, surface charge and adsorption capability), clay minerals can be used for a wide range of applications. Montmorillonite, bentonite, kaolinite and illite belong to the phyllosilicate family and are authorized as feed additives by the European Commission, and assigned to one or more functional additive groups (Table 1) (6). As an example, bentonite is an essentially impure smectite clay, mostly consisting of montmorillonite, with the ability to absorb large quantities of water and possessing a high cation exchange capacity (CEC) (7). Three types of bentonite are recognized: calcium, sodium, and potassium bentonite. Sodium and calcium bentonite are the two classes employed in the feed industry. Also tectosilicates belonging to the zeolite subfamily [e.g., clinoptilolite (CPL) of sedimentary origin] are authorized by the European Commission as feed additive (6).

**Table 1:** Characteristics of the clay minerals authorized by the European Commission as feed additives (5).

**Additive**	**Additive category**	**Additive characteristics**	**Maximum content (%) in complete feedstuff with 12% moisture**	**EU ID**
Montmorillonite-Illite	Binders Anticaking agents	≥75% phyllosilicates ≥35% montmorillonite-illite (swellable) ≥30% illite/muscovite ≤ 15% kaolinite (not swellable) ≤ 20% quartz Average 3.6% iron (structural) Free of asbestos	2	1g557
Clinoptilolite of sedimentary origin	Binders Anticaking agents	≥80% clinoptilolite (hydrate sodium calcium aluminosilicate) ≤ 20% clay minerals (free of fibers and quartz)	1	1g568
Illite-montmorillonite-kaolinite (natural mixture)	Binders Anticaking agents	≥40% illite ≥10% montmorillonite ≥8% Kaolinite Average 10% iron (structural) Asbestos free	5 (fattening poultry, ruminants, pigs; weaned piglets) 2 (other animal species)	1g599
Bentonite	Reduction of mycotoxin contamination	≥70% smectite (dioctahedral montmorillonite) <10% opal and feldspar <4% quartz and calcite BC_AfB1_ > 90%	2	1m558
Bentonite	Binders Anticaking agents	≥50% smectite (dioctahedral montmorillonite)	2	1m558i
Bentonite	Control of radionuclide contamination	≥50% smectite (dioctahedral montmorillonite)	—	1m558i

It was reported that clay minerals administered as additives in animal feed exert beneficial effects on animal physiology (8–10), although some adverse effects have been documented (11). Interestingly, in humans, geophagy is still in use in many parts of the world and specifically selected soils are consumed for medical reasons or as part of a regular diet (12, 13). Geophagy is commonly observed in animals, which are hypothesized to consume clays as a source of dietary minerals and to remove toxins possibly present in food or to treat gastrointestinal ailments (8, 9). Similarly, clay minerals have long been used by traditional medicine both as topical applications or to alleviate intestinal ailments, such as diarrhea (9, 10, 14–16).

In animal production, clay minerals are primarily used as binders for the production of pelleted feed and as adsorbents for mycotoxins and heavy metals (8–10). Indeed, diet supplementation with clay minerals, such as bentonite, is a recognized effective method to counteract the toxic effects of mycotoxins in both ruminant and monogastric species (17). Clays can also sequester phytotoxins, enterotoxins, bacteria, and viruses in the gastrointestinal tract of animals, favoring their expulsion from the body. However, each type of clay has its own specific binding capacity, and even clays from the same family may have different efficiencies against the same substance (10).

An important aspect that deserve attention, still poorly understood, is represented by the multiple ways of interaction between clays and gut microorganisms (18). This area of investigation is of the utmost importance considering the relevance of the intestinal and/or ruminal microbiota-host interactions for animal physiology (19–21). Indeed several studies suggested that the addition of different types of clays as feed additives can positively affect the performances and health of different production animals, likely by modulating intestinal and/or ruminal microbiota (chicken: (22); swine: (23); cattle: (24)). Clays can also be applied as inorganic carriers for bioactive compounds with anti-bacterial activity (25, 26) and these products may be widely employed in the future to manipulate the intestinal/ruminal microbiota.

Despite the considerable number of studies reported on the advantages of administering clay minerals for human and animal health, it should be considered that others suggested that these agents might also cause undesirable effects (10). A series of *in vitro* and *in vivo* studies documented that clay administration led to mineral and vitamin unbalances, interactions with veterinary drugs, intestinal toxicity, hepatic damage and decreased growth performances (11). In addition, anecdotal observations made by cheesemakers suggested that clay administration to cows may affect milk characteristics and cheese making properties, although, to the best of our knowledge, these observations were not supported by scientific evidences.

To further complicate the landscape, recently nanotechnologies have made available a wide range of applications in several fields and, nowadays, a wide choice of nanomaterials-based strategies is suggested to circumvent the limitations of treatments with conventional materials (27). Indeed, various types of clay nanoparticles can be produced and combined with organic polymers to produce hybrid materials, which can be used for biomedical applications (9, 28–30) and for food industry (31). Manufactured clay particles are in the ultrafine-size range with diameters ranging from few to hundreds nanometers, which, depending on their properties, can be absorbed by cells leading to modifications of viability, proliferation and functions (32). Conversely, larger clay particles (above micron size) or incorporated in polymeric products are traditionally considered as bio-inert or even biocompatible, and raise considerably less toxicity and health risk concerns (29, 33). Moreover, ingested clays are exposed to different environments (ruminal, gastric, intestinal), which may modify their characteristics and structure (11, 31, 34), thus potentially expose the intestinal cells to different clay structures.

In the near future, climate change will likely influence mycotoxin contamination of cereals and, consequently, animal feed (35). In addition, the development of strategies to limit greenhouse gas and urea emission will also be required (36–38). As clay minerals possess mycotoxin adsorptive properties and can modulate the intestinal and ruminal microbiota composition and metabolism (19, 20, 22, 24), an increased use of clay minerals in different forms is conceivable. In this context, a better understanding of the animals' physiological responses to clay mineral administration will be of the utmost importance to guarantee animal health and the safety of products of animal origin.

The present review represents a preliminary reflection for a conscious use of clay minerals in farm animals, with a special focus to ruminants. Indeed, the use of clays as feed additives in animal production deserves proper attention, considering that animal nutrition and feeding have significant implications not only on the livestock sector, but also on public health, trade, economy, and environment (36, 39, 40). Authors' aim focuses on the possible actions of different types of clay minerals not only as mycotoxin binders, but also in affecting animals' physiology. Gaps in knowledge about these topics is also highlighted to help designing the best and safest use of these feed additives.

## Structure, Classification and Physical-Chemical Characteristics of Clays

The structure and composition of different types of clay minerals have been already reviewed (10, 29, 41), and a thorough dissertation about the classification and the physical-chemical characteristics of clays is beyond the scope of the present review. In this context, it is worthwhile to provide a brief overview of the structure of clay minerals, and underline the gross differences among the different classes of clays. Authors believe that this helps the readers in understanding the different effects of clays reported in scientific literature and in suggesting to increase the awareness that an accurate description of the clay product is an important prerequisite to explain its biological effects. Differences of structure, compositions and industrial treatment clearly provide different physico-chemical properties to clays and confer different characteristics to these feed additives, which can affect the expected results (9–11, 41). As an example, among industrial treatments, commercial clays are commonly subjected to various procedures, such as contaminant removal, grinding to a finer powder and sieving to increase particle size uniformity (4).

**Figure 1:**
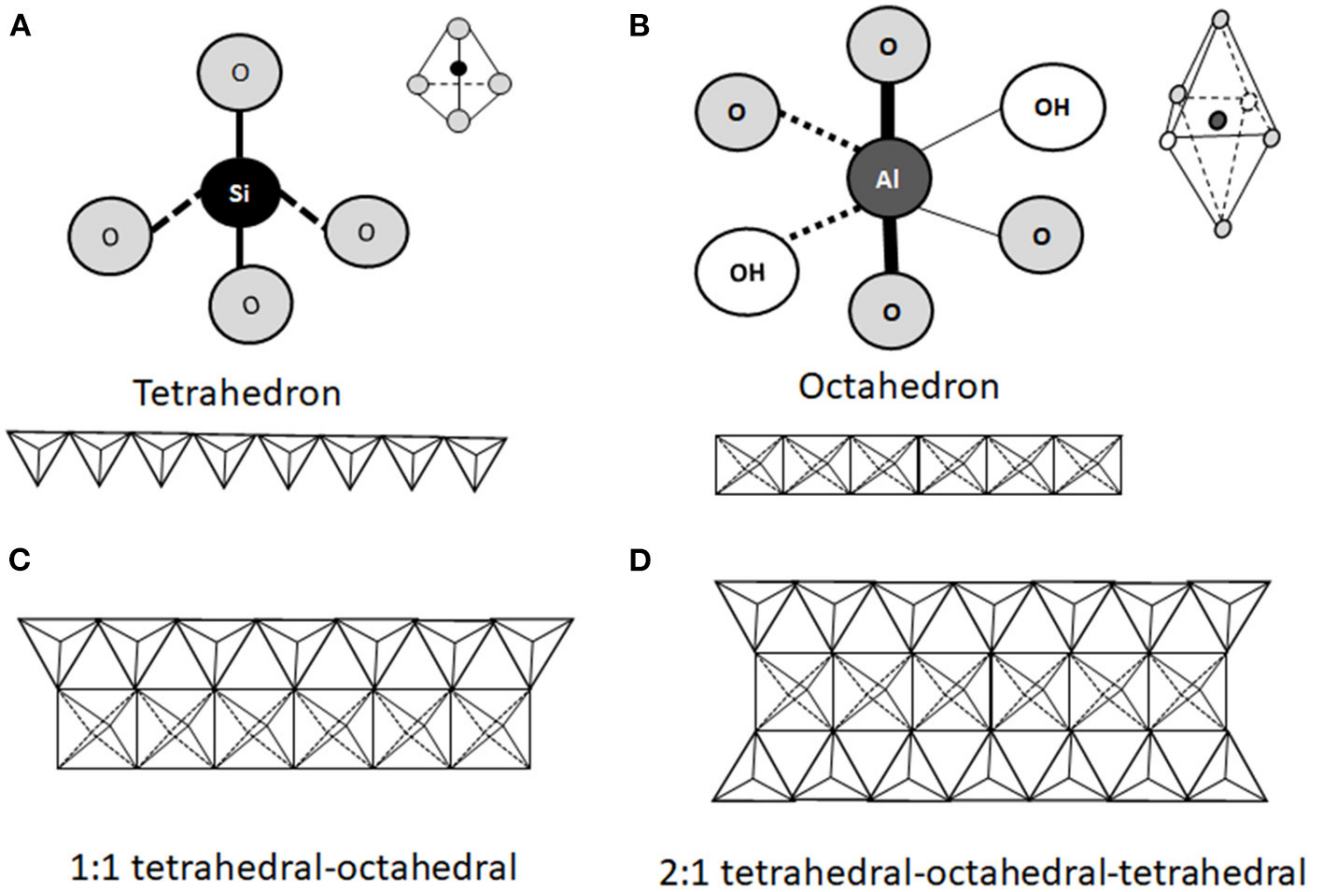
Schematic representation of the structural units of phyllosilicates. The structure consists of a combination of tetrahedral silica **(A)** and octahedral aluminum **(B)** oxide layers exposing hydroxyl groups. Two types of phyllosilicates can be distinguished. The 1:1 tetrahedral-octahedral (T-O) type consists of one layer of tetrahedral SiO_4_ joined to one octahedral aluminum (or manganese) layer **(C)**. The 2:1 tetrahedral-octahedral-tetrahedral (T-O-T) type consists in one octahedral aluminum (or manganese) layer between two tetrahedral SiO_4_ layers **(D)**.

**Figure 2:**
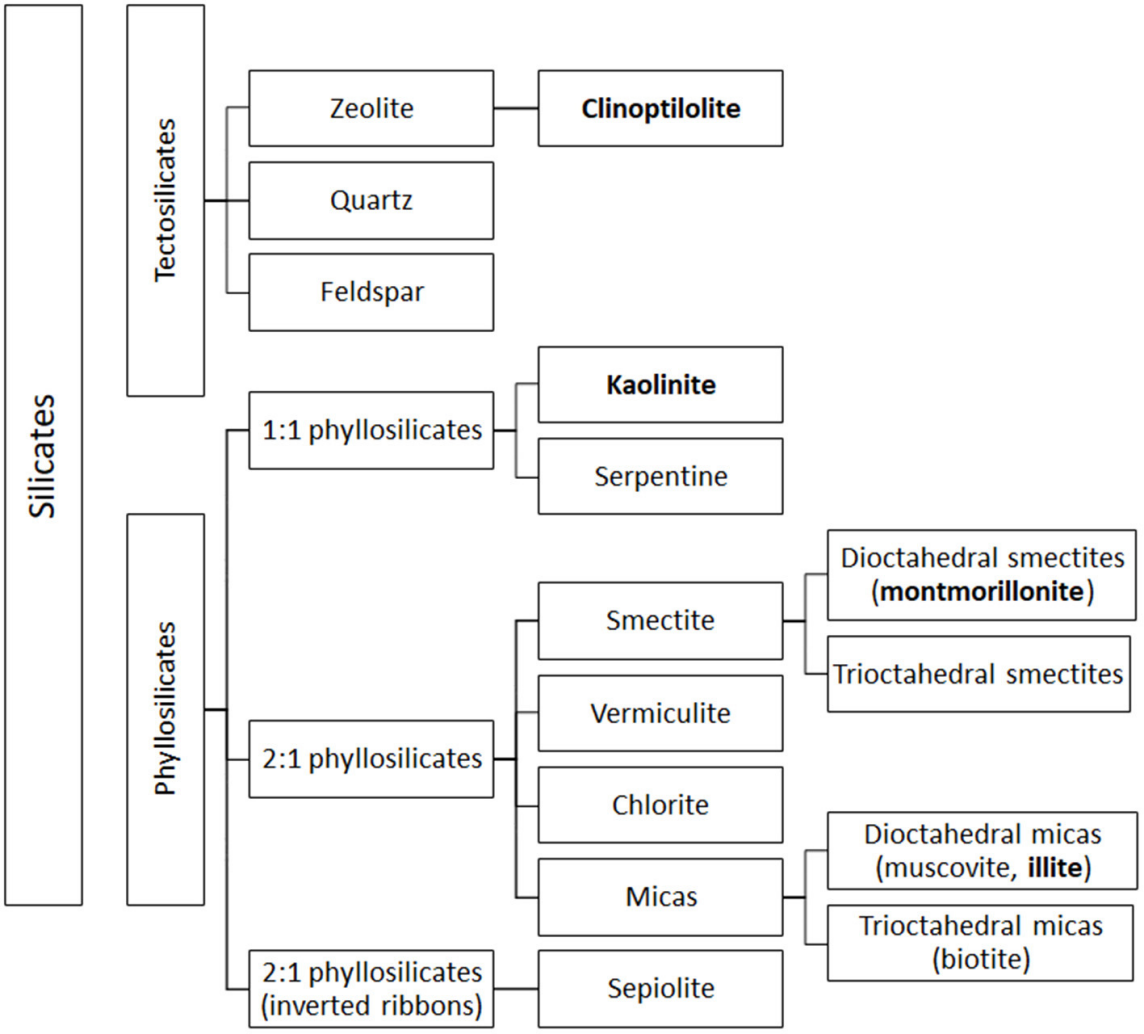
Simplified classification of silicates. Clay subfamilies and species used and present in feed additives are indicated in bold. Most clay minerals used as animal feed additives belong to the phyllosilicate family. The figure includes also the classification of tectosilicates of sedimentary origin, such as CPL, a natural zeolite that may be used as a technological additive in animal feed.

From a mineralogical point of view, clay minerals belong to the phyllosilicate family, which are characterized by parallel layers of hydrated aluminosilicates. In natural soils, clay minerals are rarely present as pure or homogeneous mixtures of single groups of minerals (42). The basic structural unit of aluminosilicate clays is a combination of tetrahedral silica and octahedral aluminum layers. The tetrahedral layers are composed of SiO_4_ units, which share three out of four oxygens (Figure 1A). The octahedral layers are composed of aluminum (or manganese) bound to oxygen and hydroxyl groups (Figure 1B). Tetrahedral and octahedral layers form two main types of phyllosilicate layers: the 1:1 tetrahedral-octahedral type (T-O; Figure 1C) consisting of one layer of tetrahedral SiO_4_ layer joined to an octahedral aluminum (or manganese) layer; the 2:1 tetrahedral-octahedral-tetrahedral type (T-O-T; Figure 1D) consisting of one octahedral aluminum (or manganese) layer between two layers of tetrahedral SiO_4_ layers (9–11). The unit layers (T-O or T-O-T) of phyllosilicates are stacked repeatedly and the distance between two adjacent layers varies depending on the type of clay. The space between the two layers, called basal spacing or interlayer, can be occupied by water and/or different ions, which confer different properties to the specific clay (41, 43). Phyllosilicate classification is based on the arrangement of the tetrahedral and octahedral layers (Figure 2). Trioctahedral layers are characterized by the occupation of divalent cations in all the octahedral sites. Conversely, in dioctahedral layers two third of the octahedra sites are occupied by trivalent cations. Under natural conditions in soils, some cations of both tetrahedral and octahedral sites are replaced by other metals of the same size even with different charge, and this phenomenon is called isomorphic substitution. In the tetrahedral sites, Si^4+^ can be partially replaced by Al^3+^ while, in the octahedral sites, Al^3+^ can be partially replaced by Mg^2+^ or Fe^2+^. Isomorphic substitution does not significantly affect the crystal structure of clays, but leads to a permanent surplus of negative charges in the aluminosilicate layers, which is balanced by the absorption of exchangeable cations, such as Mg^2+^, Ca^2+^, Na^+^, and K^+^ (3, 9, 29, 42, 43). Phyllosilicate clays have also a pH-dependent charge. Indeed, the Al and Si bound hydroxyls at the edges of clay crystals present acid-base properties and develop a negative charge at high pH and positive charge at low pH (44).

In 1:1 phyllosilicate, such as kaolinite, the strong interaction (hydrogen bonds) between neighboring octahedral and tetrahedral layers prevents water absorption and the mineral exposes only its external surface to the environment (45). Moreover, isomorphic substitution is absent, leading to low cationic exchange capacity (CEC) (1–10 cmol_c_/kg, expressed in centimole of charge per Kg), low surface area (20–50 m^2^/g) and no swelling in water (9, 28, 44). Conversely, in 2:1 phyllosilicates, isomorphic substitution accounts for most of the total charge, and the different subfamilies (Figure 2) display different degrees of isomorphic substitution, resulting in a wide range of surface charges, interlayer spaces and swelling property (3, 28, 42). For instance, in illite, belonging to mica group, the replacement of Si with 20% of Al atoms creates an important negative charge, mainly balanced by K^+^ ions, which form bridges between layers hindering mineral expansion (10, 46–48). Illite is characterized by an interlayer space of 10 Å, an intermediated surface area, and a CEC of 20–40 cmol_c_/kg (higher than kaolinite). Its adsorption and swelling capacity are lower than those of a common clay, montmorillonite (44, 47). Montmorillonite, belonging to smectite group, is characterized by a low net charge that allows the smectite platelets (the particle units) to undergo complete dissociation by osmotic swelling (28). Exchangeable cations in the basal spacing are responsible for the high expansion of the montmorillonite lattice upon hydration (42). In fact, attraction between tetrahedral sites of two neighboring layers in montmorillonite is very weak, allowing the entry of water and exchangeable cations in the crystal structure, leading to the expansion of the interlayer space (from 9.6 to 20 Å) (47). Montmorillonite is characterized by high surface area (800 m^2^/g), negative charge, high CEC (80–120 cmol_c_/kg), and high swelling ability (44, 49).

The 2:1 tetrahedral-octahedral-tetrahedral (T-O-T; Figure 1D) layers containing Na^+^ or Ca^2+^ ions represent the simple unit of the clay lattice. In naturally occurring smectite clays, several tens of layers stack together to form colloidal particles named “tactoids”, which can be leaf-like, needle-like or plate-like in shape. Tactoids display a typical lateral dimension of 100–200 nm, a layer thickness of 10 nm and an interlayer spacing of about 1 nm, and form the basic structure of clays (50–53). Tactoids can gather into different orientations and form clusters (or flocculi) displaying diameters in the sub-micrometer (nanoparticles) to micrometer range, depending on the physical-chemical conditions of the environment in which they are dispersed (54, 55). As examples, the size of illite and kaolinite particles displays a bimodal distribution showing higher frequencies at the sub-micrometer and at 1–2 μm size. The size of calcium and sodium montmorillonite particles displays a unimodal size distribution around 1–2 μm (55). A set of bentonite samples examined by European Food Safety Authority (EFSA), aimed at expressing an opinion about the safety and efficacy of bentonite as feed additive (56), was characterized by a particle size lower than 10 μm in 3–25% of the material depending on the analyzed batch. These minerals are characterized by small particle size, high porosity and high CEC, which provide the ability to react with inorganic and organic polar reagents (3). Indeed, clay nanoparticles can be produced by the modification of their layered structure, and a flourishing industry has developed various types of nanoclays (predominantly montmorillonite-based products). Moreover, nanoclays can be combined with organic polymers to produce hybrid materials, called clay nanocomposites (57), which can be used as vehicles for drugs and other bioactive substances, and for the development of biomedical applications, such as in the fields of regenerative medicine and biosensing (9, 28, 30, 41). In addition, clay nanoparticles may recite a role in food industry providing beneficial properties to food products and improved nutrition (31).

Finally, although not classified as clays, zeolites are often associated with clay minerals due to their similar properties and applications. Zeolites are silicate minerals belonging to the tectosilicate family, characterized by a 3-dimensional crystalline structure constituted of SiO_4_ and AlO_4_ tetrahedra joined by oxygen atoms to form large pores. These pores can contain weakly bound water molecules and mobile and exchangeable alkaline cations (e.g., Na^+^, K^+^, Li^+^, Ca^2+^, Mg^2+^ etc.), which balance the negative charge of the structure (10, 45, 58, 59). The properties of zeolites are related to their ability to reversibly absorb and release water, maintaining unaltered their crystalline structure, and on their structural pores, which form a kind of molecular sieve able to attract and hold positively charged atoms and molecules (10, 59–61). In some zeolites, pores can form long channels, in which ions and molecules can be easily absorbed and released (61). The best-known natural zeolite is clinoptiolite (CLP), characterized by a biocompatible nanoporous structure (26).

## Cell-Clay Particle Interactions

A thorough understanding of the interactions between clay particles and cells is important to explain both the beneficial and adverse effects of clay minerals in human and animal organisms (11, 29, 41). Clay particles as bulk materials are traditionally considered as bio-inert or even biocompatible. However, various kinds of colloidal particles with a diameter ranging from several nanometers to few microns can be taken up by cells, which can undergo changes in morphology, viability, proliferation and functions (32, 41). Most information on the interactions of clay particles with cells and on their potential cytotoxicity derives from studies performed on nanoclays (11, 28, 62–65). Other information derives from studies on clay-polymer nanocomposites, whose characteristics are very different from those of the natural clay nano or microparticles (57).

Importantly, when used as feed additives, clay minerals are ingested mainly as particles of few tens of micrometers. However, their size, structure and surface properties may change during they transit through the GI system and, to the best of our knowledge, little information is available about their fate once they are ingested. Note that, swelling and disaggregation commonly follow clays hydration. Thus, a clay mineral dispersion within an organism could be a very complex system, and may contain both individual clay mineral particles and, most likely, clay mineral aggregates. As an example, particle size of smectite materials show a multimodal distribution with components at <2 μm, primary constituted of clay mineral particles, 10–20 μm flocculi (usually resistant to disaggregation), and less common 50–500 μm micro-aggregates (55). This system is further complicated considering that the mechanical behavior of smectite in water is very sensitive to several parameters, such as its concentration, particle size and morphology, nature of exchangeable cations and chemical environment (pH and ionic strength) above all (66). Thus, within the GI system, ingested particles endure the action of physical and chemical players. Moreover, it is conceivable that clays used as feed additives release ions (34), and the particle size may be modified during the digestive processes (31). Likely, mechanical factors, such as mastication and peristalsis, should have a small impact on clay particles size. Conversely, as above mentioned, ion concentrations and pH could have a deeper effect. At acidic pH, clay minerals tend to agglomerate (31, 34). Na-montmorillonite platelets dissolve at low pH, and during this process, trivalent Al^3+^ ions are released. As a consequence, silicate platelets aggregate into tactoids (67). Conversely, at basic pH values, clay aggregates tend to disassemble to particles in the nanosize range (31, 34). In a mixed electrolyte solution, tactoid size depends also on the ratio of divalent to monovalent cations, where divalent cations (Ca^2+^) favor the formation of tactoids, while monovalent cations (Na^+^) cause a repulsion between platelets leading to aggregate disassembly (51, 68).

In humans and monogastric mammals, the pH of the stomach is definitely acidic, while intestinal pH ranges between 5 and 8 (31). In ruminants, particles reside for a considerable time interval in the reticulum-rumen, in which clays are exposed to daily pH fluctuations, ranging in dairy cows from 5.5 to 6.5 (69, 70). Thus, clays, such as bentonite, are usually administered for long time intervals as food additives for contrasting mycotoxins contamination, and clay particles possibly require a long period for being washed out from the GI tract (71), thus creating a prerequisite for their interaction with GI cells. Moreover, digestive enzymes, microbiota and interactions with biomolecules, such as bile acids, can modify particle surfaces, affecting their colloidal behavior (31) and their ability to interact with cells. Note that when nanomaterials are dispersed into biological fluids, they are rapidly coated by biomolecules (72). This coating, called corona, influence nanoparticle adhesion to the plasma membrane and, as an example, can alter cellular uptake by human adenocarcinoma alveolar basal epithelial A549 cells (73). Finally, clay nanoparticles can form a particle layer at the oil/water interface preventing coalescence of emulsion droplets, thus forming pickering emulsions (emulsion stabilized by solid particles) (74). Under simulated gastric and intestinal conditions, different clay mineral microparticles can incorporate lipids depending on their surface chemistry, thus reducing the absorption of the lipid fraction (75). Therefore, the interactions between bile acids, ingested lipids and clay minerals may play a role in modulating the effects of ingested clay mineral particles.

Once in the GI tract, and concomitantly with the above mentioned phenomena, the interactions between inorganic particles and cells depend on the cell type and on particle characteristics, and it is worth noting that size, surface properties and shape of particles play a prominent role (31, 32, 76–78). From a mechanistic point of view, clay particles interact with cell membranes by different weak bonds, such as van der Waals forces, electrostatic or hydrophobic interactions, and hydrogen bonding, but the occurrence of specific mechanisms, such as ligand-receptor interactions, could make the clay particles-cell system even more complex. The effects of particle size, shape and surface characteristics on cell viability was well described for polymeric particles (poly-lactic-co-glycolic acid or polystyrene) (79–81).

Once interacting with cell membranes, particles can be internalized by cells, being endocytosis the preferred internalization mechanism (31, 82). Inside cells, nanoparticles could cause cellular toxicity mainly by four mechanisms: reactive oxygen species production, disruption of cell membrane, induction of inflammatory response, and genotoxicity (83), and these effects can be interrelated.

In general, cells *in vitro* are able to internalize particles with a diameter lower than 10 μm and these may produce an inflammatory response. Notably, elongated and metal particles are more pro-inflammatory *in vivo* than spherical and polymeric particles (78).

As already described, the basic structural unit of clays is composed of silica and alumina layers held together by electrostatic forces. These materials, in the nano size, have large surface area and high aspect ratio (length ~2–300 nm; thickness ~1 nm), and these characteristics can confer cell toxicity (11, 63). Indeed, nanomaterials characterized by high aspect ratio can cause concerns, in comparison to isometric nanoparticles, similarly to asbestos fibers (84). Nevertheless, a recent review on the biological effects of clay nanoparticles, including nanocomposites, reported that most studies exploring nanoclay-cell interactions observed only minimal cytotoxicity in all cells tested, and, at the same time, improvements of various cell functions, such as adhesion, proliferation and differentiation (28). As an example, no genotoxic effects were reported for natural Na-montmorillonite. Specifically, according to the manufacturer, the particle size distribution of this natural clay is 10% with size <2 μm, 50% with size <6 μm and 90% with size <13 μm, and the possible content of nanometer-sized particles was not indicated (62, 85). Differently, the quaternary ammonium salt modified montmorillonite (Cloisite®30B) was moderately genotoxic on Caco-2 cells, but no clinical signs of toxicity were observed when Cloisite®30B was administered to Wistar rats (no induction of DNA strand-breaks in colon, liver and kidney cells was detected), and no increase of inflammatory cytokine markers in blood was found. Moreover, administered clay particles were not absorbed and were found in feces, thus no systemic exposure was reported (64).

Natural montmorillonite at high concentration and after long exposure time was reported to cause cytotoxic effects in human normal intestinal cells (INT-407). The cytotoxicity was probably due to the micron size of administered particles, which coated the cell surface rather than penetrating the plasma membrane. In this case, reactive oxygen species generation was also suggested to be responsible of adverse effects on cells. Moreover, no remarkable sign of toxicity was found in mice receiving up to 0.1 % montmorillonite, and no significant accumulation in any specific organ was detected, nor direct affection of *in vivo* cell viability (86). In general, “*in vitro*” and “*in vivo*” studies on clay toxicity led to controversial results. Most “*in vitro*” studies suggested that clays display different degrees of cytotoxicity through different mechanisms (necrosis/apoptosis, oxidative stress or genotoxicity). Conversely, studies performed on laboratory animals did not show clear evidences of systemic toxicity even at very high doses of clays (87).

Halloysite is a natural aluminosilicate clay with a hollow tubular structure, constituted of nanotubes of about 0.5–2 μm length and with a 10–20 nm inner luminal diameter that enables the loading and release of different molecules and drugs. Halloysite exhibits a good degree of biocompatibility in Caco-2/HT29-MTX cells under monolayer co-culture. HT29-MTX is a mucous-secreting cell line that provides a model to study the influence of the mucous layer on nanoparticle diffusion. Halloysite nanoparticles did not induce cytotoxicity despite an increased proinflammatory cytokine release (88). In the case of clay-polymer nanocomposites, it should be considered that poly-lactic-co-glycolic acid constitutes a potent inflammatory stimulus leading to NF-κB translocation to nucleus and pro-inflammatory cytokines production (89). As well, polystyrene can interact with dendritic cells depending on the surface charge of the particle (90). Furthermore, the uptake of polystyrene was described in human pro-myelocytic cell line HL60 as a function of particle hydrophobicity (91).

In the light of the above reported evidences, the possible elicitation of inflammatory response upon clay particles ingestion and interaction with GI and Gut Associated Lymphoid Tissue (GALT) cells is still an open question. Notwithstanding, the uptake of inert particles by the alimentary tract has been documented since the 1960'. It has been well established that nanoparticles can be better absorbed than microparticles (82, 92).

It should be considered that, before interacting with the cell membrane, particles have to cross the mucus layer coating the GI epithelium. The thickness and composition of mucus layers vary considerably among the different GI tract portions. In the stomach and large intestine, the mucus layer is quite thick and non-adhesive particles could diffuse through it. Conversely, the mucus layer in the small intestine is relatively thin and allows nutrient uptake by the enterocytes, while keeping at bay potentially hazardous large particles, such as bacteria (31). At the best of authors' knowledge, no specific study is available on clay micro and nanoparticles interaction with GI mucus. Generally, it could be said that pH and density of mucus have a substantial influence on this interaction and nanoparticles have more chances to cross the mucus layer than microparticles. Moreover, non-charged particles present a significant advantage in terms of diffusion in mucus (82).

Finally, immune cells, such as macrophages, can modify particle cellular uptake, and an increased intestinal permeability can result from the interaction between enterocytes and GALT. As an example, the exposure of CaCo-2 cells to THP1-derived macrophages increased the uptake of polystyrene micro-particles across the cell monolayer, likely by a macrophage-induced loosing of tight junctions and/or a decrease of epithelial depth (93). Indeed, granular pigments composed of inert inorganic particles (100–700 nm), such as kaolinite and environmental silicates, can be observed into phagolysosomes of macrophages within human GALT (94).

## Clays for Animal Production

In animal production, clays are primarily used as adsorbents to protect animals, consumers and the environment against the potentially harmful effects of feed and water contaminants (17, 49, 95–98).

**Figure 3:**
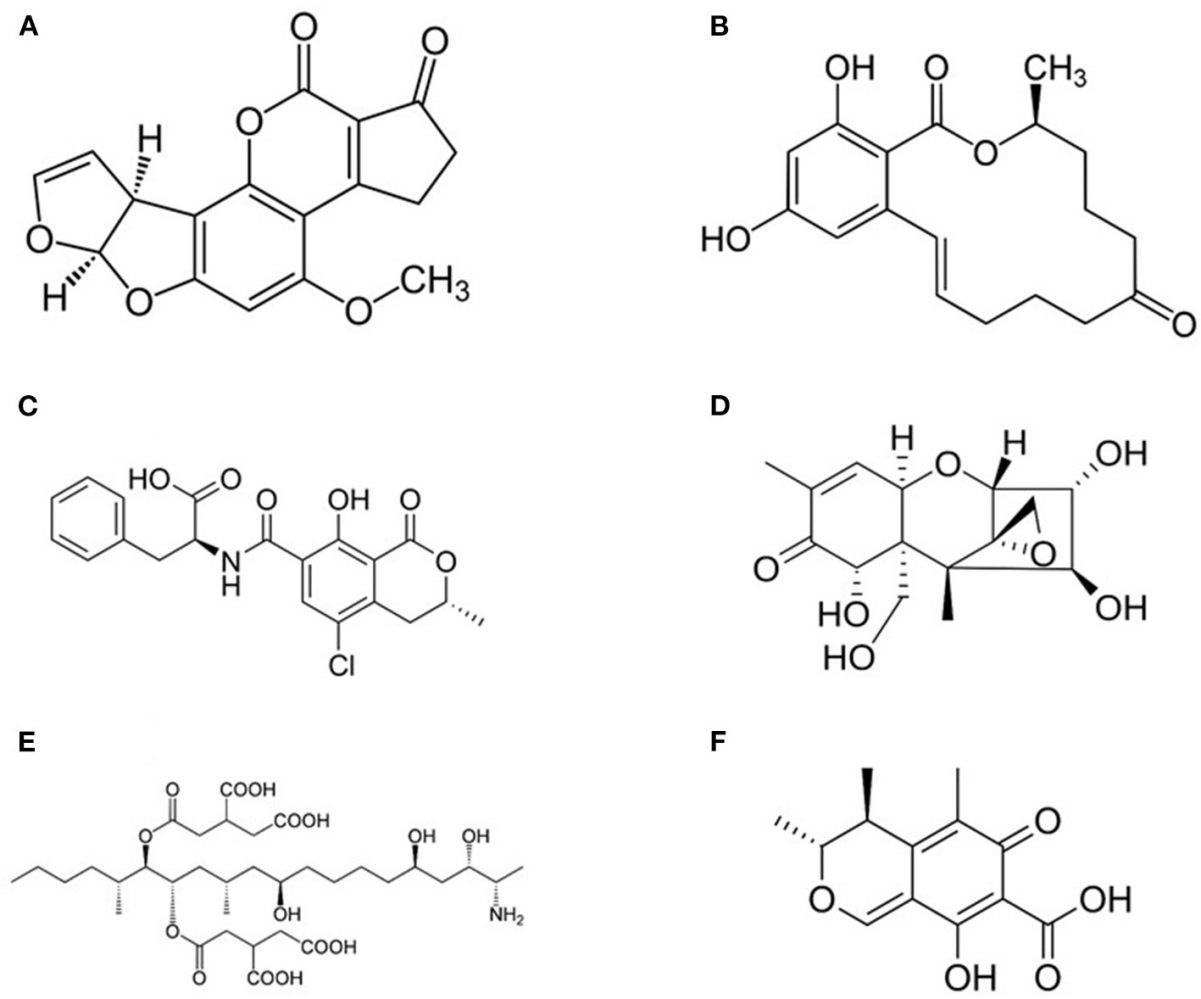
Structure of most common mycotoxins in feed. **(A)** Aflatoxin B1 (2,3,6a,9a-tetrahydro-4-methoxycyclopenta(c)furo(3',2':4,5)furo(2,3-h)(1)benzo-pyran-1, 11-dione); **(B)** Zearalenone [(3S,11E)-14,16-Dihydroxy-3-methyl-3,4,5,6,9,10-hexahydro-1H-2-benzoxacyclotetradecine-1,7(8H)-dione]; **(C)** Ochtatoxin A (N-[(3R)-5-Chloro-8-hydroxy-3-methyl-1-oxo-3,4-dihydro-1H-2-benzopyran-7-carbonyl]-L-phenylalanine); **(D)** Nivalenol [(3α,4β,7α)-12,13-epoxy-3,4,7,15-tetrahydroxy-trichothec-9-en-8-one] is a mycotoxin of the trichothecene group; **(E)** Fumonisin B1 [p(2S,2′S)-2,2′-[(5S,6R,7R,9R,11S,16R,18S,19S)-19-Amino-11,16,18-trihydroxy-5,9-dimethylicosane-6,7-diyl]bis[oxy(2-oxoethane-2,1-diyl)]dibutanedioic acid]; **(F)** Citrinin (3R,4S)-8-Hydroxy-3,4,5-trimethyl-6-oxo-4,6-dihydro-3H-2-benzopyran-7-carboxylic acid).

Mycotoxins represent one of the most challenging problem in feed and food chains and every year they appear among the “top ten” hazards reported by the Rapid Alert System for Food and Feed (RASFF) in Europe (6, 99–101). They constitute a heterogeneous group of compounds (Figure 3) produced as secondary metabolites by filamentous fungi and they are able of causing toxic effects (mycotoxicosis) in vertebrates (99, 102, 103). In farm animals, prolonged exposure to low levels of mycotoxins results in increased mortality, predisposition to infections, decreased fertility, decline of growth and weight gain, decreased milk and egg production, and, finally, higher veterinary expenses (99, 104). The carryover of mycotoxins and of their metabolites was reported in milk and dairy products, beef meat, as well as in innards and hen eggs, and in numerous tissues, organs and products from swine (17, 105, 106).

Heavy metal contamination represents another important issue all over the world. Heavy metals are elements with metallic properties belonging to different chemical groups (transition metals, metalloids, lanthanides and actinides) characterized by high atomic weight and high density (higher than 5 g/cm^3^) (107, 108). In human and animal nutrition, they have a double significance: some of them (e.g., Co, Cr, Cu, Fe, Mn, Mo, Se, and Zn) are essential micronutrients and are required in small but critical amounts for normal growth and healthy status. They act as constituents/activators of enzymes, proteins, and hormones and their deficiency can cause several symptoms, such as impaired growth, decreased fertility and immune defense, anemia, neurological disorders, ataxia and skeletal deformities (108, 109). Other heavy metals are generally considered as non-essential so-called xenobiotics as they do not seem to play key roles within organisms, or can negatively affect the level of essential elements in the body (108). Therefore, non-essential heavy metals are undesirable substances in animal feed. In addition, some of them (Al, As, Cd, Hg, Ni, Pb) display toxic and carcinogenic properties even at very low concentration (97, 110–112). Furthermore, heavy metals are subjected to bioaccumulation and biomagnification along the food chain. The carryover of heavy metals is reported in many animal tissues and organs and in products for human consumption (i.e. milk, eggs, meat) (97).

Clay minerals can adsorb mycotoxins, heavy metals and other toxic compounds (e.g., phytotoxins, diarrheagenic enterotoxins, harmful microorganisms and radionuclides) by different mechanisms (Table 2) (26, 48, 79–82). Mycotoxin sequestration involves different types of molecular interactions. Physical interactions are generally reversible and influenced by environmental conditions (pH, ionic strength) and include hydrophobic bonds, such as Van der Waals forces, and electrostatic forces, such as dipole-dipole interactions. Typically, these interactions are frequently involved in mycotoxin absorption by clays. Differently, chemical binding is irreversible and occurs when adsorbent and adsorbate form a covalent interaction. The adsorption efficiency depends on the characteristics of both the clay (e.g., pore density, size and distribution, electric charge) and the mycotoxin (e.g., polarity, shape, size and solubility) (124). In general, because of their hydrophilic and negatively charged surfaces, aluminosilicates are more effective in binding polar mycotoxins, such as aflatoxins (AFs), than the less hydrophilic zearalenones (ZEAs), ochratoxin A (OTA), and trichotrecenes (TCTs) (59, 124). Negatively charged clays are also poorly effective against acidic mycotoxins, such as fumosins (FUMs), which in solution tend to form anionic species by deprotonation of –COOH groups (125). Studies on the mechanism of interaction between clays and mycotoxins have focused mainly on smectites and AFs and different adsorption models have been proposed (Table 2).

**Table 2:** Adsorption mechanisms of AFB_1_ by raw mineral adsorbents determined by *in vitro* trials (113).

**Mineral adsorbent**		**AFB_**1**_ concentration (mg/Kg)**	**Experimental conditions**	**Adsorption efficacy mg/Kg)**	**Adsorption mechanism**	**References**
**Type**	**Concentration (%)**					
Hydrated sodium calcium aluminosilicate (HSCAS)	Not reported	Not reported	Not reported	131,000^a^	Selective chemisorption by mononuclear bidentate chelation	Phillips et al., (114)
Ca-montmorillonite	0.5	0.0–2.0	Incubation in water (pH 2.0 or 8.0) at 37 °C for 1.5 h under intermittent mixing	613.5^b^ at pH 2 628.9^b^ at pH 8	Hydrogen bonds on the edges of Ca-montmorillonite	Desheng et al., (113)
Ca-montmorillonite	Not reported	Not reported	Not reported	Not reported	Electron donor acceptor (EDA) on the negatively-charged surface	Phillips et al., (115)
Smectite clays	2.0	0.0–8.0	Incubation in water under shaking for 24 h	18,000–212,000^b^	Coordination bonds, hydrogen bonds with smectite interlayer cations or associated water molecules	Kannewischer et al., (116); Tenorio Arvide et al., (117)
Ca-smectite	0.03	33.3^c^	Incubation in water under shaking overnight	140,000^b^	Hydrogen bonds with hydration shells of exchangeable cations or coordination with exchangeable cations	Deng et al., (118)
Ca-montmorillonite	0.1	1.0–2.0	Phosphate-buffered solutions at pH 3.5 (gastric conditions), 6.5 (intestinal conditions) and 9 37 °C under shaking for 60 min	50^b^	Ion-dipole interactions and coordination with exchangeable cations	Wang et al., (119)
Smectite	1.0	3–10	Incubation in water at 25°C for 3 days under shaking	3–400	Strong EDA coordination via Ca^2+^-bridging on the surface	Kang et al., (120)
Illite	1.0	3–20		3–300	A moderate EDA attraction by the negatively charged surface sites	
Kaolinite	1.0	5–30		1–150	Weak H bonding	
Natural zeolite	0.25	0–2	Simulated human digestion solutions	Not reported	Sorption on the external surface	Albayrak et al., (121)
Zeolite	1.5	0.1	Digestion model simulating dynamic gastrointestinal tract of poultry	4.7	Binding mechanisms not determined; possibly electrostatic attractions, EDA attraction, and calcium-bridging linkages	Zavala-Franco et al., (122)
Zeolite	0.5	0.1	Digestion model simulating dynamic gastrointestinal tract of avian species	15.1^d^	Binding mechanisms not determined; possibly electrostatic attractions, EDA attraction, and calcium-bridging linkages	Vázquez-Durán et al., (123)

a *Maximum binding capacity (B_max_)*;

b *Maximum adsorption (q_max_)*;

c *Exposure of smectite to aflatoxin repeated twice*;

d *Maximum adsorption capacity*.

The ability of clays to adsorb heavy metals mainly depends on their CEC and on their specific surface area. In general, clay minerals characterized by high porosity (ex. smectites) have a higher surface area than those exposing only the external surface to the solvent (ex. kaolinite, illite) (59, 126, 127).

Studies on animals (mice, pig and fish) suggested that zeolites, mainly CLP, can be proposed as useful feed additives for the prevention of intoxications by heavy metals. However, the *in vivo* effectiveness of clays as heavy metal absorbent should be further in depth assessed. Indeed, the interactions between clays and heavy metals depend on several factors, such as contact time, clay dosage, pH of GI tract, temperature, the presence of other metal species or of organic substances which could affect the adsorption capacity (126, 128, 129). In addition, it is important to bear in mind that clays can even release metal ions (e.g., Al^3+^) or adsorb essential microelements (e.g., Zn, Cu, and Mg), potentially causing mineral imbalances (11).

The adsorption capacity and/or the affinity of clays towards mycotoxins and heavy metals can be improved by various treatments. For example, the replacement of inorganic with organic cations (usually quaternary ammonium compounds) leads to the formation of organo-clays, which are characterized by weaker interlayer interactions leading to increasing surface area and reduced hydrophilicity (49, 119, 126, 130). Alternatively, calcination (heat treatment at temperature from 600 to 1,000°C) leads to the production of thermally modified clays, characterized by a reduced mass, increased porosity and available surface for ion exchange (126). Moreover, clay minerals can be subjected to acid activation by treatment with hydrochloric, phosphoric, nitric or sulphuric acid, which modifies the material surface and removes cationic impurities, opening pores and edges, increasing available binding sites (126). Smectite minerals can also be exposed to soda activation using sodium carbonate, by which the swelling and adsorption proprieties can be increased (131).

In dairy cows, several clay mineral preparations can be successfully used as mycotoxin absorbents, as they are effective in reducing aflatoxin M1 (AFM1) excretion in milk (60, 132–139). In this view, Na-bentonite seems more effective than Ca-bentonite, probably due to its higher swelling capacity and Na to Ca ratio, that increases the surface area and cation exchange (60). The administration of aluminosilicates to dairy ruminants exposed to aflatoxin containing diets resulted in a corresponding dose-dependent reduction of aflatoxin excretion in milk, urine and feces (133–137).

The improvement of productivity and of some patho-physiological indicators can be viewed as indirect evidences of the positive effects of clay administration. For example, clay minerals can promote growth performance in pigs and broilers, and egg production in hens (26, 45, 49, 140). In pigs, clay administration can successfully counteract the negative effects of AFs and zearalenone by preserving or even improving feed intake, feed efficiency, weight gain, growing performance, and serum clinical chemistry profile (141, 142). Different results have been observed after CPL inclusion in the diet of piglets (59). As reported, CPL caused a significant improvement of feed conversion in the period from weaning to slaughter. Differently, other authors did not record any improvement of pigs growth and feed conversion rate upon CPL administration with feed (143). Interestingly, the same authors suggested that CPL was effective as immunomodulatory agent by promoting the recruitment of circulating and intestinal lymphoid cells (143, 144). Experiments on laying hens reported that the addition of CPL in the diet increased the number of eggs, shell thickness and the efficiency of feed utilization (145). Other authors observed on the same animal a significant increase of Al and Zn concentrations in serum, possibly related to the improved quality of egg shell and bone development (146). This may be explained by the partial solubilisation of clays (zeolites) leading to the release of their structural elements, even if the process should not be feasible at the physiological pH of the animal gastrointestinal tract (45). In broiler chickens, CPL supplementation resulted in an increased animal body weight, and in an improvement of organoleptic meat parameters and in ω-3 polyunsaturated fatty acid levels (147). Beneficial effects of aluminosilicate supplementation on performances and health status have been observed also in poultry receiving aflatoxin or ochratoxin contaminated diet, while conflicting results were obtained in case of toxin T-2 contamination (113, 148–152). In ruminants, aluminosilicate administration led to the improvement of mycotoxin-related health conditions, such as oxidative stress and liver inflammation and damage (133, 137). However, controversial effects on milk yield and quality were observed when AFs were administered with clays, as some authors referred positive effects (133, 139), while others did not report any effect (132, 134–136) or even a reduction of milk yield and quality (137). In sheep, bentonite administration favored wool growth, which is sensitive to amino acid availability. It is therefore conceivable that bentonite is responsible of a reduction of ruminal protein degradation (153). The capability of bentonite to prevent metabolic disorders, to increase microbial protein production and to improve rumen pH and fermentation conditions has been considered for explaining the improvement of weight gain and feed consumption efficiency in steers, although high amount of clays was reported to cause mineral deficiencies due to their high binding capacity (154). In dairy heifers, the long-term inclusion of CPL in the diet improved the energy status, milk production and reproductive parameters, possibly due to beneficial effects on ruminal and/or post-ruminal digestion of starch (155). In addition, the supplementation of both natural and modified CPL in cow feeding has been observed to improve the energy status, the reproductive performances and to reduce the intramammary infections postpartum (156–159). Dietary CPL influenced also the blood levels of Ca and P in dairy cows, improving the serum Ca:P ratio during the early post-partum period (160). The reproductive performances were positively affected by the ingestion of mineral adsorbents in cattle (161) and sows (162) fed with AFB1 contaminated total mixed ration. In addition, clay administration displayed positive effects to contrast production diseases, such as milk fever and ketosis, due to the ability to reduce Ca availability in the gastrointestinal tract and to improve the energy balance (59). Bentonite was effective in reducing the incidence, but not the severity, of bloat in dairy cows (163). Clay administration may also alleviate the effects of subacute ruminal acidosis (SARA), leading to an increase of ruminal and fecal pH and to the modification of rumen volatile fatty acids. In particular, an increase in acetate and a decrease in propionate and valerate were observed, along with an increased milk yield, milk fat and energy content (164, 165).

## Effects of Clays on the Gi Physiology

In the present review, we focused our attention mainly on the potential interactions between clay minerals and cells of the GI system and the GALT.

In this view, the ability of clay minerals to absorb feed and water contaminants, as well as endogenous produced toxins, may account for most of their beneficial effects. However, there are also indications that some of the positive effects of clays on the production performances of farm animals probably depend on their ability to increase nutrient utilization and on positive effects on intestinal physiology, documented both in humans and animals, even though the underlying mechanisms of action are not fully understood.

### Effects of Clays on Nutrient Digestibility

Several mechanisms may account for the positive effects of clay supplementation on the growth performances observed in swine and poultry (166–169). An important feature of clay minerals is represented by the mobility of chemical elements in their interlayers vacancies when they are exposed to environments with different physical-chemical characteristics, such as through the gastro-intestinal (GI) tract. Indeed, clays contain different elements, such as Na, K, Mg, Fe, and Ca, which may play particular biochemical roles in improving body weight and feed conversion rate (166). In this view, clay nanoparticles present chemical and physical properties completely different from those of large-scale (micro) particles, in particular regarding their surface to mass ratio, making ion exchange much more available (170). As an example, Na^+^, commonly found in nanoclay minerals, is involved in many fundamental cellular functions, such as acid base balance and the absorption of amino acids (AA) and glucose (171).

Thus, clays are potential sources of dietary minerals, either beneficial or potentially dangerous, but the presence of specific minerals in soils or ingested bulk materials does not necessarily guarantee any effect in term of mineral availability. The possible mineral supply obtained by eating clays depends on mineral composition and physicochemical properties, in particular, on CEC of the ingested material (13). Specifically, gastric and intestinal environments may play a role in mineral mobility of clays.

The effects of chemical leaching during geophagic clay digestion were examined in a series of experiments simulating the gastric and intestinal environments. Although far from being conclusive, results suggested that both beneficial and dangerous chemical elements could be released from clays during digestion (34).

Clay nanoparticles can also increase the intestinal uptake of other nutrients by slowing down the transit rate of the intestinal content due to the formation of gels that increase the feed viscosity (172). As a consequence, GI enzymes can be more effective on nutrient digestion (172). Moreover, an increase of the secretion and of activity of digestive enzymes were reported in both swine and poultry, respectively, after zeolite or sepiolite supplementation (168, 173). Different mechanisms were proposed: while zeolite affects intestinal pH, resulting in higher digestive enzyme secretion and activity (168), sepiolite forms stable aggregates with pancreatic enzymes, which remain active in a wider range of pH (173).

The effects of clays on six different kinds of feedstuff were tested in an *in situ* digestion experiment. Clay supplementation altered the degradability of grass hay, wet brewer's grains, soybean meal, and corn silage, and increased nutrient digestible fractions of feedstuffs thanks to their improved degradation, probably due to alteration of rumen microbial population (174). An increase of digestibility of dry matter, organic matter, crude protein, ether extract, non-fiber carbohydrates and neutral detergent fibers was observed also in lactating goats during clay supplementation when fed with a diet naturally contaminated with aflatoxin B1 and zearalenone. However, the study did not established whether the improved nutrient utilization observed following the administration of clay minerals was an effect of mycotoxin neutralization or a direct effect on the rumen fermentations (139). In sheep, improved nutrient digestibility was observed when bentonite was administered at 4% of the diet (175, 176).

### Effects of Clays on Gastro-Intestinal Mucosa

Clay administration was observed to influence the intestinal mucosa morphology and function in several animal models.

Morphological modifications and overexpression of proteins involved in lipid metabolism were observed in the enterocyte brush border of rats having free access to kaolinite during refeeding (177). Kaolinite supplemented rats showed an increase in the thickness of the villi with large vacuoles at the base of the mucosal cells and a decrease of the enterocyte microvilli length. Moreover, modifications of the expression level of cytoskeleton proteins was evidenced by proteomic analyses of intestinal mucosa. Few dissociated kaolinite particles and aluminum originating from the ingested clay were observed in the intestinal lumen and within the mucus barrier. Interestingly, aluminum could directly cross the intestinal mucosa and this should be further investigated, considering the potential neurotoxicity of Al (178).

Diosmectite, a natural silicate used for the treatment of infectious diarrhea, can adsorb toxins and bacteria, and modify the rheological characteristics of gastrointestinal mucus. Diosmectite administration showed anti-inflammatory activity, a general amelioration of the intestinal epithelium morphology, of biomarkers of oxidative stress and a modulatory action of cytokine production by mucosal cells (14). In pigs, in comparison with untreated animals, CPL administration resulted in a higher fraction of lymphoid cell subsets, with exception of CD8+ T cells, and higher recruitment of CD45RA+ (a marker for memory T cells) cells in interfollicular, but not in follicular areas of the ileum Payer's patches (143).

Clay administration demonstrated protective effects on the intestinal mucosa by physical reinforcing the mucous barrier, the first line of defense during infections, which is possibly responsible for the documented antidiarrheal and anti-inflammatory effects of clays (15, 179). The slower transit rate of intestinal content, the increase of enzyme activity and morphological changes of the enteric mucosa are among the causes of the reduction of incidence, severity and duration of diarrhea (15, 140). A contribution to clay anti-diarrheal effects may come from illite and smectite adsorptive properties, which cause a reduction of water and cation enteric excretion leading to the increase in the fecal consistence (180). In weaned piglets, dietary supplementation with the hydrous magnesium-aluminum silicate palygorskite (a synonym for attapulgite) improved growth performance and reduced the incidence of diarrhea. Palygorskite administration increased the intestinal villus diameter and lymphocyte number in the jejunum and resulted beneficial to the intestinal integrity (181). Authors suggested that palygorskite might exert its protective action by forming a protective screen on mucosa layer. The protective action of diosmectite on mucus was observed in rats (182, 183), where diosmectite binds to mucin reducing the pepsin-induced mucolysis induced by inflammation. In this case, diosmectite may be included in the adherent intestinal mucus (15). In addition, an increase in the number and size of mucin-producing goblet cells was observed in pigs fed with smectite (180).

Clay minerals have also been examined as potential tool against SARA. Clay supplementation in cows suffering from SARA increased rumination and pre-stomach pH, reduced blood lactate concentration and modulated the concentration of liver enzymes (24, 165). These positive effects can be explained by clay buffering effect, related to their H^+^ adsorption capacity (184), in the GI tract, and by the slowing of the transit rate from the rumen. This may influence the rumen and intestinal microbiota, favoring the production of acetate, and reducing propionate in the rumen, as well as the amount of fermentable carbohydrates in the intestine for post-ruminal fermentations (164, 165). Moreover, it was reported that clay supplementation influenced the concentration of several AA and biogenic amines (BA) depending on the presence of specific ruminal bacteria (24). The presence of BA in systemic circulation have deleterious effects on animal health, while AA are essential for the metabolism and immune system. Therefore, clay-dependent modifications in rumen microbiome can improve the maintenance of integrity of nonspecific defenses of the gut wall (185). The enhancement of the defensive capacity of the mucosal immune system has been also observed in chicken supplemented by zeolite and attapulgite (palygorskite) by an increase of antioxidant capability and antibacterial activity, as well as of the concentration of secretory immunoglobulin A in jejune mucosa (167). Finally, effects of clays on the modulation of cytokine production were observed, and anti-inflammatory properties were evidenced (14).

These results suggest that clays can interact with the ruminal and intestinal microbiome (19), although further investigations are needed to clarify the interconnections.

### Effects of Clays on Microbiota

Clays can interact with microorganisms in multiple ways (18), and many of the effects of clays in animals may result from the modulation of the intestinal and/or ruminal microbiota (19, 22, 23, 71, 186). Moreover, clay minerals display antibacterial properties that could be exploited as a potential alternative to the use of antibiotics (8, 49, 187, 188). Indeed, montmorillonite can alter the permeability of bacterial cellular membranes allowing the diffusion of intracellular ions and low molecular-weight metabolites (25, 189). As a practical example, the increased growth and elevated organoleptic characteristics of meat in broilers receiving CPL were associated with reduced total gut microbiota and enteric infections (147). Moreover, the antibacterial properties of clays may be selective. Montmorillonite administration can counteract reduced nutrient digestibility, increased oxidative stress and reduced growth observed in weaning gilts receiving zearalenone. Montmorillonite was beneficial for detoxification of zearalenone, possibly by the selective modification of the intestinal microbiome, resulting in an improved *Lactobacillus* population and a decreased *E. coli* count (23). In another study, montmorillonite supplementation to pigs promoted the growth of *Lactobacillus* and *Bifidobacterium* at the expense of *Clostridium* and *E. coli* (179). In sheep, bentonite administration impaired protozoa viability, leading to the reduction in ruminal degradation of feed protein and bacteria predation (153).

The ion exchange capacity of montmorillonite can be responsible of modifications of pH and oxidative state of the intestinal environment, favoring some bacteria over others. Montmorillonite could also damage the bacterial cell membrane and enhance the adsorption of bacterial toxins (179). The hydrogen bonding between diosmectite and enterotoxins of *E. coli, V. cholerae* and *Clostridium* species prevented the interactions of the toxins to cellular membrane receptors, preventing mucosa damage (190–192). Montmorillonite may also facilitate the flush out of gram-negative bacteria from the intestine, rather than inhibiting bacteria growth. In fact, montmorillonite can adsorb *E. coli* cells on its surface (25) by anchoring bacteria to the positive charged sites on clay surface (193), or by the cell adhesion through the fimbriae located in their wall (25).

Clays can modulate the ruminal microbiome also by regulating ammonia availability, as these minerals can reversibly adsorb this molecule as a function of its concentration, functioning as an ammonia buffering system for ruminal microbiome (139, 175). When rumen microbes are not efficient in capturing ammonia, this molecule is absorbed and converted into urea in the animal liver, and then excreted with urine. When urinary urea enters in the environment, it breaks down to ammonia and nitrous oxide as environmental pollutants (194). In this context, bentonite administration could be seen as a feeding management strategy to reduce nitrogen wastage. Thus, mineral adsorbents can be seen as an animal feed and slurry management strategy against the environmental dispersion of nitrogen by reducing ruminal ammonia release and improving its utilization by ruminants, and reducing ammonia volatilization from slurries (45, 165, 195, 196).

## Potential Contraindications of the Use of Clays

So far, the actions of clays against the negative effects and the carryover of mycotoxins and the positive side effects of clays on some production parameters of farm animals have been highlighted. However, it is important to bear in mind that not all studies reported positive effects of clays, which in some cases were ineffective or even responsible of an increase of mycotoxicosis symptoms or of other negative side effects (11). For instance, according to Meisinger and colleagues most of the experiments on the ability of sequestering agents to detoxify fusarium toxins carried out *in vivo* in swine and poultry did not demonstrate any preventive effect (197).

Due to their non-specific mechanism(s) of action, clay minerals can interact with substances different from mycotoxins. Their effectiveness depends on their type and concentration and on the specific mycotoxin tested, but it is also influenced by other factors, such as feed composition, presence of specific ions and molecules (including proteins, enzymes and vitamins) and pH (198, 199). Indeed, clay minerals are able to interact with metabolites and nutrients, such as nucleic acids, AA and proteins by cation exchange, electrostatic interactions, hydrophobic/hydrophilic interactions, hydrogen bonding and van der Waals forces (200, 201). These interactions are commonly considered beneficial, as they are related to a higher amount of bypass proteins and in the more efficient utilization of AA in ruminants (202). The possible application of clays as vectors for gene and drug delivery was also suggested (201). However, clay minerals can be responsible for imbalances of essential nutrients, for possible interactions with hormones and veterinary drugs, leading to negative effects on animal health and productive parameters (11). *In vitro* experiments simulating the ingestion of geophagic soils/clay minerals demonstrated that these additives can reduce the bioavailability of some essential elements, such as Fe, Cu, and Zn (203, 204), and can enhance the bioavailability of others, such as Ca, Mg and Mn (203). A series of experiments in rats fed with a diet supplemented with 2% bentonite showed that this clay improved BW gain and stimulated Fe absorption, although causing a moderate persistent decrease of Ca and Se absorption and of their organ content (205–207). Despite the detected deficiencies reported in these studies were moderate, it must be considered that Se intake is marginal strongly depend on geographical region (207). However, other studies in rats did not find any relevant alteration following the dietary administration of processed calcium montmorillonite (up to 2%), neither in pregnant animals (208) nor after long treatments (28 days) (209, 210). Also the inclusion of up to 2% Na-bentonite in rat diet did not show signs of toxicity even if administered for 3 months (211). Regarding farm animals, (212, 213) reported a reduction of Mn availability in poultry treated with 0.5% bentonite and (214) noted that 0.5% sodium montmorillonite was responsible of a decrease in P serum content in broiler chickens. In contrast, diet supplementation with 0.5 and 1.0% hydrated sodium calcium alumino-silicate (HSCAS) in chicks did not compromise the utilization of Mn, phytate and inorganic P, as well as of vitamin A and riboflavin, but caused a slight decrease of Zn utilization (79, 215). Excessive administration of bentonite (≥ 2%) in chicks can induce poor weight gain and obvious signs of vitamin A deficiency up to an increase in mortality (by 4 weeks administration of 5% bentonite) (216). Moreover, sodium bentonite affected testosterone and thyroid hormone levels in male broiler chicks (217). Diet supplementation with zeolite in laying hens could be harmful for the formation of the eggshell, since 1, 2, and 3% administration resulted in increased Zn and Al levels and decreased Mg and Cu concentrations in serum (218). However, (219) did not observe any considerable alteration of egg quality due to dietary administration of CPL (2%) in laying hens. Excessive administration of montmorillonite can also impair growth performance and health in starter pigs: inclusion of more than 1% in the ration had negative effects on liver structure and serum mineral concentrations and 5% montmorillonite caused a decrease in feed intake, aggravation of liver damage and a reduced antioxidant capacity (220). In female weaned piglets, a diet with 0.4 % aluminosilicate failed to counteract the effects of *Fusarium* toxins, tended to reduce feed intake and feed to gain ratio, decreased serum concentrations of cholesterol and α-tocopherol, increased levels of albumin, aspartate transaminase, and γ-glutamyl transferase, but it did not affect the concentrations of retinol and retinyl esters in liver and serum (221).

Undesirable effects of clay mineral administration have also been reported in ruminants. In rams, bentonite decreased the ruminal availability of Cu, Zn, and Mg and the liver concentrations of Cu and Mg (153). However, considering the predisposition of sheep to Cu poisoning, the reduction of dietary Cu bioavailability could be seen as a positive effect of bentonite (153). Feeding growing goats with diet supplemented with zeolite at 0.12 and 0.16 % for 3 weeks, (80) recorded slight alterations of serum concentration and excretion of Ca, signs of increased bone resorption without alterations of bone structure. The treatment led also to the significant decline in plasma concentrations of P, Mg, and 1,25-dihydroxycholecalciferol and in renal excretion of P, warning of possible negative long-term effects. The addition of micronized zeolite up to 2% to lamb feed did not affect animal performance and carcass yield, but affected serum total protein, calcium and phosphorus concentrations. Increasing zeolite dose to 3%, led to a decrease of slaughter weight, hot and cold carcass weights (81). Bentonite administration at 5 and 10% in Holstein cow fed with high-grain ration was responsible of a significant decrease of energy and crude protein digestibility and of a statistically significant decrease of Mg and P (222). However, lower concentrations of bentonite (0.125, 0.25, 0.5, and 1%) were sufficient to counteract AFs effects and did not alter dairy cows' status and production (bodyweight, body condition score (BCS), dry matter intake, milk yield, milk quality and composition, minerals, vitamin A and riboflavin concentrations in milk) (134–136).

In humans, 2 weeks oral treatment with 1.5 or 3.0 g/die of NovaSil caused in the first 2 days gastrointestinal symptoms (bloating, constipation, diarrhea, flatulence, and abdominal pain) in 24 and 28% volunteers, respectively, and dizziness in two subjects receiving 1.5 g NovaSil. However, no statistical significance was found of these adverse effects between the two groups. After treatment, statistically significant decrease (within the normal range of clinical references) in blood levels of RBCs, hemoglobin, total protein, albumin, ALT, and S were recorded in the low-dose group, but not in the high-dose group. A significant dose-dependent increase of serum Sr was observed. No other significant differences in hematology, liver and kidney function, and in electrolyte, mineral, vitamins A and E concentrations were found in both groups. According to authors, results suggest the relative safety of NovaSil clay in humans (223).

Regarding the interactions between adsorbing agents and drugs/pharmaceuticals, binding phenomena to clays can enhance or reduce the effects of drugs (11, 212, 213). For example, tylosin was ineffective in cattle when bentonite was concomitantly administered (224) and led to reduced or even canceled efficacy of tilmicosin in poultry (225). Devreese et al. demonstrated, by providing tylosin in broiler chickens, that bentonite was able to bind the macrolide antibiotic altering its pharmacokinetics (226). Moreover, bentonite can reduce the effectiveness of coccidiostats, such as monensin and salinomycin (213, 227, 228).

Altogether, results here reported evidence that further studies are need to investigate on interactions between clays and biological relevant nutrients and drugs. These evidences should suggest the adoption of specific controls and interventions in animal health practice, modifying animal diet, dosages and withdrawal times of drugs and/or adsorbent additives, in order to prevent cases of toxicity or nutrient deficiency/antibiotic ineffectiveness (related to possible enhancement of microbial resistance development) in animals and to safeguard public health. EFSA has already proposed to ban the simultaneous use of coccidiostats when bentonite is administered above 0.5 % and recommended to report the information on the label of bentonite packaging to avoid its oral use concomitantly with certain medicinal substances (e.g., macrolides) (212). Analogously, Food Drug Administration (229–231) has ruled to eliminate the use of robenidine, ipronidazole, and buquinolate in combination bentonite.

## Conclusions and Future Perspectives

The use of clay minerals in the agro-food sector seems destined to increase for several reasons: the first is the adoption of prevention and decontamination strategies able to minimize food and feed contamination by mycotoxins and, consequently, reduce health and economic risks for humans and animals. Likely, foreseen climate changes will affect mycotoxin production and distribution in different world regions and these toxins are predicted to become a major problem in Europe within the next 100 years (232). Indeed, climate will become milder in northern countries, making these areas suitable for fungal growth and mycotoxin production. At the same time, heat and drought in southern countries will cause a decrease in agricultural production up to desertification and a replacement of currently prevalent mycotoxins with AFs (35, 233–235).

Heavy metals are naturally present in soil and atmosphere, fresh and salty waters. Moreover, human activities (e.g., mining, industrial production, waste dumping, etc.) significantly contribute to heavy metal pollution (107, 108). In addition, certain agricultural and farming activities can be considered either as a source of heavy metals or can contribute to their recirculation (97, 108). The administration of clay minerals can be applied for controlling heavy metals absorption by farm animals.

Finally, an increased use of clay minerals can be expected for their putative contribution to fight against various problems linked to intensive farming (e.g., antibiotic resistance, SARA, greenhouse gases and ammonia emission, etc.), which represent a huge economic problem for farmers and involve the consumers' sensitivity toward issues, such as animal wellbeing and environmental sustainability.

National and international authorities set the maximum limits or guide levels for the use of clays and clay minerals as feed additives, which may be an ideal choice given their physical-chemical properties, low cost, low or null toxicity and eco-compatibility. However, it must be considered that clay minerals are not completely inert additives, and can interfere with intestinal/ruminal metabolism with possible consequences on animal health. Most studies on clay minerals, aimed at evaluating their effectiveness against toxic compounds, paid poor attention toward any possible nonspecific collateral effect. Furthermore, many studies did not report in detail the characteristics of the clay used, making the interpretation of results difficult. Other studies examined mostly productive parameters, which, as well, may not be sufficient to provide an exhaustive picture of the possible unwanted effects of clays on animal health and physiology (11, 197). Therefore, further studies are needed, particularly on ruminants, to verify possible interferences of clays with rumen fermentations and metabolite uptake, which may affect animal metabolism and, possibly, milk characteristics. In particular, future studies should consider the effects of long-term administration/accumulation of clays. Finally, the fate of clay particles during their transit within the GI system should be analyzed as, although clay micro and nanosize particles seem not to be cytotoxic at the intestinal level, their capacity to stimulate an inflammatory response should be carefully considered and their effects on already diseased subjects should be taken into account.

## Author Contributions

AD has planned and drafted this review as a part of her PhD project. All authors listed have made a substantial, direct, and intellectual contribution to the work and approved it for publication.

## Funding

This review is partially funded by the Italian Ministry of Agricultural, Food and Forestry Policies (MIPAAF, DM 27443−25/09/2018, project BENFELAT). AD benefitted of a PhD grant from the University of Padova.

## Conflict of Interest

The authors declare that the research was conducted in the absence of any commercial or financial relationships that could be construed as a potential conflict of interest.

## Publisher's Note

All claims expressed in this article are solely those of the authors and do not necessarily represent those of their affiliated organizations, or those of the publisher, the editors and the reviewers. Any product that may be evaluated in this article, or claim that may be made by its manufacturer, is not guaranteed or endorsed by the publisher.

## Abbreviations

AA: amio acids
AFs: Aflatoxins
CEC: cationic exchange capacity
CPL: clinoptilolite
GALT: Gut Associated Lymphoid Tissue
GI: gastrointestinal
SARA: subacute ruminal acidosis.
